# The role of immune regulation in peripheral nerve regeneration: functions of inflammatory cells and cytokines

**DOI:** 10.3389/fphar.2026.1735833

**Published:** 2026-03-04

**Authors:** Yongkun Zhang, Haochun Zhang, Yun Su, Min Nuo, Wenguang Wu, Haochen Jiang, Xiangjun Meng

**Affiliations:** 1 Department of Orthopaedics, Affiliated Zhongshan Hospital of Dalian University, Dalian, Liaoning, China; 2 Department of Ophthalmology, Affiliated Zhongshan Hospital, Dalian University, Dalian, Liaoning, China

**Keywords:** cytokines, immune regulation, inflammation, macrophage polarization, peripheral nerve regeneration, Schwann cells, signaling pathways

## Abstract

**Background:**

The regenerative repair following peripheral nerve injury is a complex pathophysiological process in which the immune regulatory network plays a crucial role. Conventional understanding posits that inflammatory responses impede nerve regeneration; however, recent studies reveal that immune reactions constitute a “double-edged sword”: a well-timed and moderate inflammatory response is essential for initiating regeneration, whereas excessive or persistent inflammation deteriorates the regenerative microenvironment and hampers repair. This review systematically elaborates the dynamic responses of the innate and adaptive immune systems after peripheral nerve injury. We focus particularly on the phenotypic switch of macrophages from the pro-inflammatory M1 to the anti-inflammatory/reparative M2 type, the early debris-clearing function of neutrophils, the interactions between T lymphocytes and Schwann cells, as well as the intricate signaling networks formed by cytokines and chemokines. The article delves into how these immune cells and factors precisely regulate key processes in Schwann cells—such as dedifferentiation, proliferation, migration, and myelination-thereby influencing axonal regeneration and functional recovery. Finally, this review prospects the translational potential of optimizing the immune microenvironment by targeting specific immune cells or signaling pathways for treating peripheral nerve injuries. Deciphering these delicate immune regulatory mechanisms will provide a critical theoretical foundation for developing novel immunomodulatory strategies to enhance nerve regeneration.

**Summary:**

In this review, we outline current understanding of the immune mechanisms underlying peripheral nerve regeneration, spanning from established paradigms to emerging therapeutic approaches, such as targeted immunomodulation, biomaterial-assisted microenvironment reshaping, and adoptive immune cell therapy, all of which represent promising avenues for improving functional recovery after nerve injury.

**Key Messages:**

The integration of immunology and nerve regeneration research is opening new frontiers for treatment. Harnessing the regenerative potential of the immune system while restraining its detrimental effects—through approaches such as precision modulation of macrophage polarization, neutrophil extracellular trap regulation, and T cell-Schwann cell crosstalk targeting—offers encouraging prospects for overcoming the current challenges in peripheral nerve repair.

## 1 Introduction

Peripheral nerve injury represents a common cause of disability in clinical practice, and its repair and regeneration constitute a complex pathophysiological process involving the coordinated action of multiple cells and factors. For a long time, the inflammatory response was widely regarded as a negative factor impeding repair in the field of nerve regeneration (Zhai et al., 2025). However, with advances in neuroimmunology, it is increasingly recognized that the immune system plays a more refined and dynamic regulatory role in shaping the microenvironment following neural damage.

Recent breakthroughs have revealed that the immune response exhibits distinct spatiotemporal specificity and dual regulatory characteristics (Li et al., 2025). In the early phase after injury, a well-modulated inflammatory response is not only necessary for clearing necrotic tissue but also acts as a critical switch for initiating the intrinsic repair program (Zhang et al., 2022). Immune cells directly participate in core processes such as Schwann cell phenotypic reprogramming, axonal guidance, and angiogenesis through the secretion of cytokines and growth factors, thereby providing structural support and nutritional foundations for nerve regeneration (Min et al., 2021). However, when the inflammatory response becomes dysregulated or persistent, its effects undergo a fundamental reversal, leading to neuronal apoptosis, glial scar formation, and collapse of the neurotrophic support system, ultimately causing irreversible damage to the regenerative microenvironment (Poletti et al., 2024).

The elucidation of this “double-edged sword” nature has shifted the research paradigm in nerve regeneration from traditional “anti-inflammatory” strategies towards a new focus on the “precise modulation of the immune microenvironment” (Sun et al., 2021). The current research emphasis is no longer confined to simply suppressing inflammation but is aimed at deciphering the dynamic dialogue between immune cells and neural cells, and exploring how to coordinate pro- and anti-inflammatory effects within specific spatiotemporal windows to maximize the constructive potential of the immune system (Tian et al., 2021). This article will begin with the bidirectional regulatory role of inflammation to systematically elaborate the core regulatory network involving immune cells (such as macrophages, T lymphocytes) and their secreted factors in nerve regeneration (Xie et al., 2024). It will further delve into their complex manifestations in conditions like neuropathic pain and diabetic peripheral neuropathy. Furthermore, the review will assess emerging therapeutic strategies targeting immune regulation, including pharmacological interventions, biomaterials, and the multi-targeted approaches of Traditional Chinese Medicine. It will analyze current challenges in clinical translation and propose future research directions for nerve repair based on remodeling the immune microenvironment. By comprehensively carding the intrinsic connections between the immune system and nerve regeneration from multiple dimensions, we aim to provide a theoretical basis and innovative ideas for developing temporally controlled and precise immunomodulatory therapies.

## 2 Methods/literature search strategy

This narrative review was conducted to synthesize and critically evaluate the current understanding of immune regulation in peripheral nerve regeneration. A comprehensive literature search was performed using the PubMed, Web of Science, and Scopus databases for articles published from January 2000 to March 2025. The search employed the following key terms and their Boolean combinations: (“peripheral nerve injury” OR “nerve regeneration”) AND (“inflammation” OR “neuroinflammation”) AND (“macrophage” OR “Schwann cell” OR “cytokine” OR “chemokine”) AND (“immune modulation” OR “therapy*”). Inclusion criteria encompassed original research articles (*in vitro*, *in vivo*) and high-impact reviews focused on molecular mechanisms, cellular interactions, and therapeutic interventions related to inflammation and immunity in peripheral nerve injury (PNI). Exclusion criteria were non-English articles and studies solely on central nervous system injury without peripheral nerve relevance. Relevant articles were thematically analyzed to construct the narrative framework of this review, which is structured to first elucidate fundamental mechanisms (Sections 1–3) and then discuss their translational implications and therapeutic targeting (Sections 4–5).

## 3 The dual role of inflammation in peripheral nerve regeneration: from mechanisms to therapeutic interventions

### 3.1 The beneficial role of inflammation: constructing a regenerative microenvironment

The inflammatory cascade following peripheral nerve injury is initiated by the immediate release of Damage-Associated Molecular Patterns (DAMPs) from ruptured axons, degenerating myelin, and compromised Schwann cells (Lin et al., 2025). These endogenous “danger signals,” which include molecules like high-mobility group box 1 (HMGB1), heat shock proteins (HSPs), ATP, and DNA fragments, are recognized by pattern recognition receptors (PRRs) - such as Toll-like receptors (TLRs) and receptors for advanced glycation end products (RAGE) - expressed on resident immune cells (e.g., endometrial macrophages) and Schwann cells themselves (Ramadan et al., 2017). This DAMP-PRR interaction serves as the critical “alarm signal,” triggering the activation of key transcription factors like nuclear factor kappa-B (NF-κB) and activator protein 1 (AP-1). Subsequently, this leads to the rapid production and secretion of pro-inflammatory cytokines and chemokines (Rubartelli and Lotze, 2007). The resulting chemokine gradient is responsible for the precise spatiotemporal recruitment of circulating neutrophils and monocytes to the injury site, marking the transition from a sterile insult to an active immune response. Thus, DAMP-mediated signaling is not merely a bystander effect of injury; it is the fundamental mechanism that orchestrates the initial immune cell infiltration, setting the stage for the subsequent dual-phase (destructive and constructive) roles of inflammation in nerve repair.

During the initial phase of peripheral nerve injury, a well-modulated inflammatory response serves as a critical driver for initiating and ensuring successful regeneration. At this stage, inflammation first performs a debridement function: neutrophils and macrophages that rapidly infiltrate the injury site clear necrotic tissue along with degenerated axonal and myelin debris through efficient phagocytosis, thereby creating a pristine microenvironment for subsequent axonal regeneration (Jin et al., 2012). Subsequently, inflammatory signals initiate the repair program: pro-inflammatory cytokines such as TNF-α and IL-1β, released by immune cells, activate signaling pathways including NF-κB within Schwann cells, driving their phenotypic switch-from a mature myelin-maintaining state to an active repair state through dedifferentiation (Guo et al., 2024). This transition is characterized by the downregulation of myelin-associated genes and the upregulation of repair-related genes such as c-Jun and glial cell line-derived neurotrophic factor (GDNF), prompting Schwann cells to proliferate and align into critical structures known as Bands of Büngner, which guide directional axonal extension (Li et al., 2019; McAlhany et al., 2000; Sangaraju et al., 2019; Vaudano et al., 2001). Notably, under specific conditions and at low concentrations during the early injury phase, cytokines like IL-1β and IL-6 can directly promote Schwann cell proliferation and enhance their ability to synthesize and secrete neurotrophic factors such as nerve growth factor (NGF), exerting beneficial neuroprotective and pro-regenerative effects in the short term (Chen et al., 2016; Zhou et al., 2020). Furthermore, to meet the high energy demands of the regeneration process, vascular endothelial growth factor (VEGF) secreted by macrophages and fibroblast growth factor-2 (FGF-2) synergize with angiopoietin-1 released by Schwann cells to effectively promote neovascularization at the injury site (Duobles et al., 2008; Ganta et al., 2019; Huang et al., 2014). This nascent vascular network supplies essential oxygen and glucose to regenerating neurons and Schwann cells, thereby providing robust metabolic support for nerve regeneration (Keilhoff et al., 2008; Tang et al., 2023).

### 3.2 The detrimental role of inflammation: chronicity and regenerative failure

When the inflammatory response becomes uncontrolled or transitions from acute to chronic, its nature shifts from constructive to destructive, severely impeding the repair and regeneration of peripheral nerves through multiple interconnected mechanisms (Cobo et al., 2018; Nguyen et al., 2023; Vanucci-Bacqué and Bedos-Belval, 2021). First, the persistent inflammatory microenvironment exerts direct neurotoxicity (Cao et al., 2024). Sustained high levels of TNF-α engage the TNF Receptor 1 (TNFR1) on neurons, leading to the assembly of the caspase-8 activating complex (DISC), which initiates the extrinsic apoptotic cascade. Similarly, IL-1β can impair mitochondrial function, promoting cytochrome c release and activation of the intrinsic caspase-9 apoptotic pathway (Ng et al., 2018; Tylutka et al., 2024; Wang and He, 2018; Kim et al., 2004; Trichonas et al., 2010; Beckham et al., 2010; Hu et al., 2019). Second, a severe imbalance occurs in the regulation of immune cells, which is central to the repair process (Hua et al., 2019; Li et al., 2022). The sustained polarization of macrophages towards the pro-inflammatory M1 phenotype results in excessive secretion of detrimental factors like IL-6, TNF-α, and matrix metalloproteinase-9 (MMP-9) (Davis et al., 2018; Han et al., 2025). This not only suppresses the reparative functions of Schwann cells but also disrupts the structure of the Büngner bands (Roet et al., 2014), which are essential for guiding axonal growth. Concurrently, the anti-inflammatory and pro-repair M2 macrophage phenotype is suppressed, preventing the timely resolution of inflammation and creating a vicious cycle (Li P. et al., 2024; Lopes et al., 2024; McKenzie et al., 2006). Third, dysregulated inflammation drives detrimental tissue remodeling (Antonangeli et al., 2020). Excessive activation of fibroblasts, driven by factors like TGF-β1 (Montero et al., 2021), leads to the production of abundant collagen fibers and inhibitory molecules such as chondroitin sulfate proteoglycans (CSPGs) (Li et al., 2021; Zhang et al., 2024). The CSPGs in the scar tissue primarily exert their inhibitory effect by binding to the Protein Tyrosine Phosphatase Sigma (PTPσ) receptor on growth cones. This interaction leads to the inactivation of integrin signaling and, most critically, the activation of the small GTPase RhoA and its effector ROCK (Rho-associated kinase). RhoA/ROCK activation then drives actomyosin contraction, causing growth cone collapse and halting axonal advancement (Jessen et al., 2015; Joosten et al., 2000; Li et al., 2015). It is noteworthy that endogenous compensatory mechanisms exist to counteract this inhibitory signaling. For instance, neurons can upregulate receptors like the LAR family phosphatases, which may compete for CSPG binding (Xu et al., 2015), or activate counteracting pathways *via* neurotrophic factors (e.g., BDNF, GDNF) (Cornejo et al., 2021). These factors can signal through PI3K/Akt to inhibit RhoA activity and promote the activity of growth-promoting GTPases like Rac1 and Cdc42. However, in a chronic inflammatory milieu characteristic of conditions like diabetic neuropathy, these protective pathways are often suppressed or overwhelmed, allowing the inhibitory CSPG-RhoA/ROCK axis to dominate and contributing to regenerative failure. Furthermore, chronic inflammation impairs the crucial neurotrophic support system (Gurnani et al., 2025). On one hand, it downregulates the ability of Schwann cells to synthesize neurotrophic factors like Nerve Growth Factor (NGF) and Brain-Derived Neurotrophic Factor (BDNF) (Meyer et al., 1992). On the other hand, it causes desensitization of the corresponding receptors on neuronal surfaces (Zhang et al., 2009). Consequently, regenerating neurons are deprived of external trophic support and become unable to effectively respond to these supportive signals (Masson and Nait-Oumesmar, 2023; Titz et al., 2003), plunging them into a state of “starvation” and increasing their risk of apoptosis (Fan et al., 2021; Previtali, 2021). Finally, inflammation disrupts the structural foundation maintaining the neural internal environment. Overproduction of factors like Vascular Endothelial Growth Factor (VEGF) increases the permeability of the blood-nerve barrier, leading to substantial infiltration of inflammatory cells from the vasculature and accumulation of inflammatory exudates. This causes local edema, which compresses nerve fibers and further deteriorates the already vulnerable regenerative microenvironment (Suter et al., 1993; Wu et al., 2022).

**FIGURE 1:**
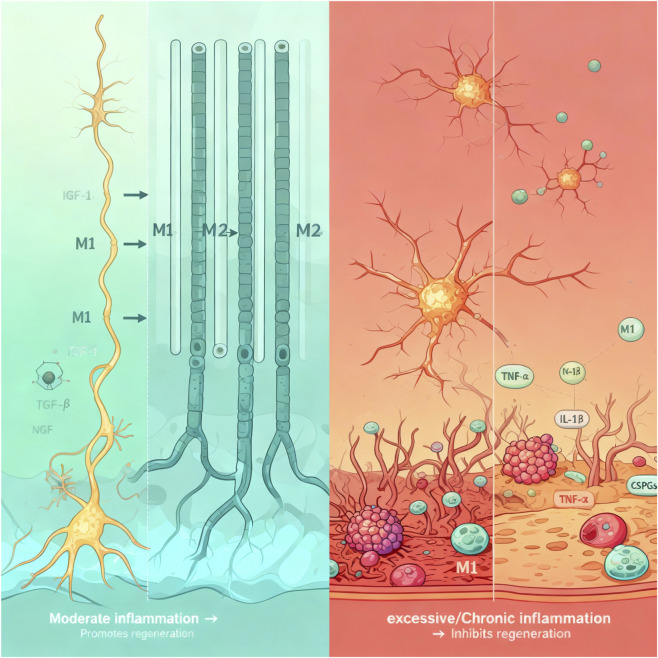
Schematic diagram of the dual role of inflammation in peripheral nerve regeneration. The left panel illustrates how moderate inflammation promotes regeneration by clearing debris, facilitating the reparative phenotype of Schwann cells, and stimulating angiogenesis. The right panel demonstrates how excessive/chronic inflammation impedes regeneration through mechanisms including direct neurotoxicity, disruption of the blood-nerve barrier, and inhibition of neurotrophic support.

In summary, inflammation plays a spatiotemporally dependent dual role in peripheral nerve regeneration, and its precise regulation is pivotal to the success of regeneration. As illustrated in Figure 1 above, this “double-edged sword” effect persists throughout the entire process.

## 4 Core regulatory functions of immune cells in nerve regeneration

### 4.1 Phenotypic switching and functional regulation of macrophages

An appropriate degree of inflammation is an essential component of functional recovery following neural injury (Munro et al., 2012). However, excessive inflammation leads to persistent activation of immune cells and degeneration of neural cells (Gao et al., 2022; Zhang et al., 2010). Spatiotemporal modulation of M1/M2 macrophage polarization can reshape the local inflammatory immune microenvironment, steering it toward a state conducive to tissue repair (Van den Bossche et al., 2016). Following peripheral nerve injury, macrophages precisely regulate the regenerative microenvironment through the secretion of various factors (Gu et al., 2024; Li X. et al., 2024; Wang JY. et al., 2024). Macrophages precisely regulate the regenerative microenvironment through secreted factors that activate specific cellular programs. For instance, macrophage-derived IL-6 binds to the IL-6 receptor on Schwann cells, activating the intracellular JAK/STAT3 signaling pathway. This phosphorylation cascade directly translocates to the nucleus to upregulate genes such as Cyclin D1, thereby driving Schwann cell cycle entry and proliferation (Leibinger et al., 2016). Conversely, TGF-β secreted by reparative macrophages primarily signals through the Smad2/3 pathway in fibroblasts and Schwann cells. This activation leads to the transcription of genes encoding extracellular matrix (ECM) components like collagen I and fibronectin, but crucially, in a regulated manner that promotes healthy ECM remodeling rather than dense fibrotic scarring (Huang et al., 2024). Furthermore, IGF-1 exerts its pro-regenerative effects by binding to the IGF-1 receptor on neurons, which activates the PI3K/Akt/mTOR survival pathway, inhibiting apoptosis and promoting axonal outgrowth. Research utilizing electrospinning techniques has constructed a multifunctional, multi-layered nanofiber composite membrane from polycaprolactone (PCL) and amniotic membrane (AM) (Xue et al., 2019). *In vitro* studies demonstrate that the PCL/AM composite promotes axonal growth in SH-SY5Y cells and induces their differentiation into neurons (Liu et al., 2023). When used to wrap nerve stumps, the PCL/AM composite creates a microenvironment favorable for nerve regeneration by blocking the invasion of scar tissue, promoting macrophage recruitment and their moderate polarization toward the M2 phenotype (Lu et al., 2024). This process enhances the expression of anti-inflammatory cytokines IL-10 and IL-13 while suppressing the expression of pro-inflammatory cytokines IL-6 and TNF-α, ultimately inducing myelination and axonal regeneration (Abidi et al., 2022). By releasing various bioactive substances that modulate M2 macrophage polarization and the formation of anti-inflammatory factors, the PCL/AM complex can enhance axonal regeneration and improve neurological repair (Wang et al., 2019).

### 4.2 Interactions between immune cells and Schwann cells

Under physiological conditions, Schwann cells in the peripheral nerve exist primarily in two functional subtypes: myelinating Schwann cells, which ensheath large-diameter axons to form the myelin sheath, and non-myelinating Remak Schwann cells, which envelop multiple small-diameter axons. Following peripheral nerve injury, both subtypes undergo a process of dedifferentiation, downregulating myelin-maintaining genes (e.g., MPZ, PMP22) and upregulating a repair-specific program. This transforms them into a plastic, proliferative “repair” phenotype. Inflammatory signals following peripheral nerve injury serve as the key initiating factor driving the phenotypic switch of Schwann cells from a myelinating state to a repair state (Jiang et al., 2025). Pro-inflammatory cytokines such as TNF-α and IL-1β activate pathways like NF-κB, which downregulate the expression of myelin-associated genes (e.g., MPZ, PMP22) in Schwann cells while upregulating repair-related genes (e.g., c-Jun, GDNF) (Huang et al., 2015). This dedifferentiation enables Schwann cells to proliferate extensively and align into critical structures called Büngner bands, which guide axonal regeneration (Schuh et al., 2016). These cells also secrete extracellular matrix components such as laminin and fibronectin, providing essential “tracks” and support for regenerating axons.

Activated Schwann cells further contribute by secreting neurotrophic factors like NGF and BDNF, which activate the PI3K/Akt survival pathway in neurons, alongside releasing GDNF, which is crucial for motor neuron regeneration (Bierlein De la Rosa et al., 2017). Together, these actions form a favorable intercellular signaling network that supports regeneration. However, this repair process operates within a critical time window; persistent or excessive inflammatory signals can lead to Schwann cell senescence or tissue fibrosis, ultimately impeding nerve regeneration (Cutolo et al., 2022).

## 5 Complex manifestations of neuroimmune interactions in pain and disease

### 5.1 Immune mechanisms of neuropathic pain

Peripheral nerve injury and disease often lead to persistent pain that continues after the initial injury has subsided, indicating an active disease process that may result in chronic pain conditions (Sommer et al., 2018). While well-controlled neuroinflammation can promote regeneration and healing, impaired resolution of neuroinflammation can lead to chronic pain (Subhramanyam et al., 2019). Research over the past decades has accumulated substantial knowledge about these physiological and pathophysiological processes and identified potential therapeutic targets (Bishop et al., 2021). Key participants in inflammatory processes include macrophages, T lymphocytes, cytokines, and chemokines (Cassol et al., 2015). Within the spinal cord and brain, microglia and astrocytes actively contribute to disease progression. MicroRNAs and other non-coding RNAs have been identified as potential key mediators linking neural injury, pain, and inflammation (Daidone et al., 2021). Among the clinical conditions most extensively studied in the context of neuroinflammation and pain are complex regional pain syndrome, polyneuropathy, postherpetic neuralgia, and fibromyalgia syndrome (Sommer et al., 2018; Wen et al., 2023). Studies from several research groups have demonstrated that both pro-inflammatory and anti-inflammatory cytokines play significant roles in human neuropathic and other chronic pain states. Substantial evidence indicates that anti-inflammatory cytokines exert analgesic effects in animal models (Herrerias et al., 2009). The interaction between anti-inflammatory cytokines and the nociceptive system presents both opportunities and challenges for therapeutic development.

### 5.2 Challenges and complexities in clinical research

After animal models clearly demonstrated the critical role of cytokine-mediated neuroimmune responses in pain, research focus naturally shifted toward clinical validation. However, establishing a specific link between cytokines and pain symptoms in patients with neuropathy has proven far more challenging than anticipated. Early studies targeting circulating cytokines initially appeared to support the “pain susceptibility” cytokine profile hypothesis. A prospective study found that compared to patients with painless neuropathy and healthy controls, patients with painful neuropathy exhibited significantly elevated mRNA and protein levels of pro-inflammatory cytokines (e.g., IL-2, TNF-α) in their blood, while painless patients showed a predominant upregulation of the anti-inflammatory cytokine IL-10 (Table 1) (Baka et al., 2021). Notably, this difference was independent of the etiology of the neuropathy (e.g., whether it was an inflammatory neuropathy), suggesting the potential existence of a cross-disease, endogenously determined immune imbalance state that predisposes individuals to pain susceptibility (Meacham et al., 2017). A similar trend was partially corroborated in studies on painful diabetic neuropathy (Zhang et al., 2021). Nonetheless, the results from these blood-based studies have been inconsistent, potentially limited by patient cohort heterogeneity, sample size, and variations in detection methodologies.

**TABLE 1:** Example of a comparative study on cytokine profiles between patients with painful and painless neuropathy.

Study sample	Measurement indicators	Painful neuropathy vs. painless	Potential implications and limitations
Peripheral blood	IL-2, TNF-α	↑↑ (mRNA and protein)	It suggests a correlation with a systemic pro-inflammatory state; however, this can be confounded by systemic factors
​	IL-10	↓ (mRNA)	Anti-inflammatory capacity may contribute to the occurrence of pain
​	IL-4	↑ (protein)	The alteration patterns are complex and require further investigation
Lesioned tissues (nerve/Skin)	IL-6, IL-10	↑ (mRNA)	The changes in the local microenvironment are not entirely consistent with the blood results
​	Neurotrophic factors (e.g., BDNF, NGF)	↓ (mRNA)	Nerve repair-supportive environment is a shared feature of neuropathy, rather than a pain-specific one
​	T Cell/Macrophage infiltration	No direct correlation	This finding challenges the oversimplified view that the degree of infiltration directly dictates pain intensity

To more precisely elucidate pain mechanisms, subsequent studies shifted to direct analysis of affected sural nerve and skin samples. However, the results were unexpected: local tissue cytokine changes proved far more complex than those in blood (Kong et al., 2022). More importantly, the severity of neuropathic pain showed no direct correlation with the extent of T-cell or macrophage infiltration in nerve or skin tissues. These negative findings suggest that neuropathic pain may not be simply determined by the absolute levels of a few cytokines, but rather stems from dysregulation within a more intricate and dynamic network of local cellular interactions (Baron et al., 2010). A key insight is that neuropathy involves extensive alterations in cutaneous gene expression, indicating that pain maintenance may rely on sustained interactions between nerve endings and non-neuronal cells (e.g., keratinocytes, immune cells) within the skin (Kreß et al., 2021).

### 5.3 The role of angiogenesis and metabolic support in nerve regeneration

**FIGURE 2:**
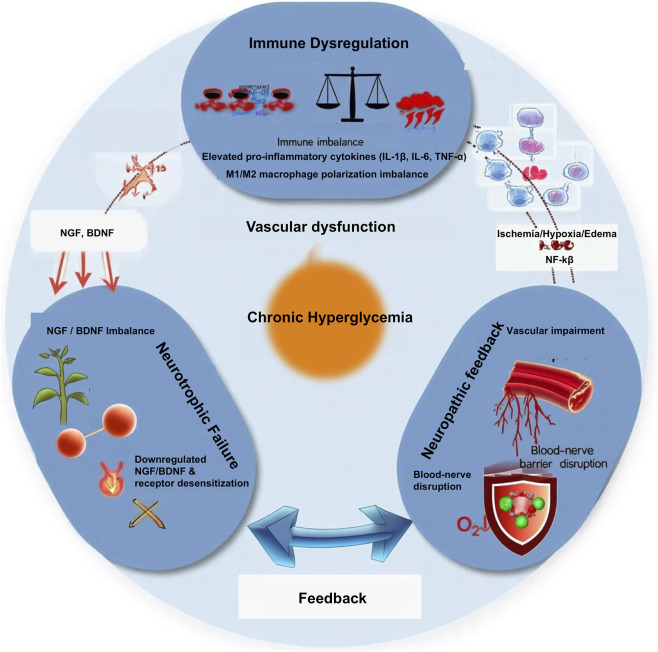
The vicious cycle mechanism in DPN. Chronic hyperglycemia drives three core pathological processes: immune-inflammatory imbalance, vascular support dysfunction, and neurotrophic failure. These three components mutually reinforce each other, forming a self-sustaining vicious cycle that leads to failure of nerve regeneration.

As illustrated in Figure 2, under the pathological conditions of diabetic peripheral neuropathy (DPN), this precisely orchestrated repair program is severely disrupted by the chronic inflammatory microenvironment resulting from persistent hyperglycemia (Greten and Grivennikov, 2019; Stone et al., 2017). Prolonged metabolic dysregulation constitutively activates canonical inflammatory signaling pathways, such as nuclear factor kappa-B (NF-κB), *via* pattern recognition receptors (e.g., Toll-like receptors) (Hilgendorf et al., 2024). This not only directly triggers neuronal axonal degeneration and myelin breakdown (Wallerian degeneration) but also leads to sustained high-level expression of pro-inflammatory cytokines (e.g., IL-1β, IL-6, TNF-α), establishing a persistent inflammatory cascade that is difficult to resolve (Monk et al., 2015). These cytokines play a particularly complex dual role in DPN, with their effects being highly dependent on the specific spatiotemporal context and concentration. On one hand, they exacerbate neural damage, insulin resistance, and pathological pain through direct neurotoxicity (e.g., IL-1β inhibiting the PI3K/Akt neuronal survival pathway, TNF-α inducing apoptosis) and by indirectly activating glial cells to produce more inflammatory mediators (Hyung et al., 2018; Sugiura and Lin, 2011). On the other hand, under specific conditions, inflammatory signals are necessary for initiating repair; for instance, IL-1 has been shown to transiently promote Schwann cell proliferation and upregulate nerve growth factor (NGF) expression, suggesting its potential to switch from a damaging factor to a reparative signal (Hughes and Appel, 2016; Salzer, 2015). Once this delicate balance is disrupted by chronic inflammation, it ultimately compromises the neurotrophic factor system, which is vital for neuronal survival and functional maintenance.

Neurotrophic factors (e.g., NGF, NT-3) play a central role in maintaining neuronal soma health, guiding directional axonal growth, and promoting myelination (Ricci et al., 2024). In the DPN state, the chronic inflammatory microenvironment can lead to significant downregulation of neurotrophic factors and their receptors, severely impairing their anti-apoptotic, pro-growth, and Schwann cell-supporting functions, thereby fundamentally constraining effective nerve regeneration and repair (Peng et al., 2023).

In summary, the pathogenesis of DPN can be viewed as a vicious cycle network comprising three interconnected components: vascular support dysfunction, immune-inflammatory imbalance, and neurotrophic failure. A deep understanding of the spatiotemporal dynamics and interactions of the various factors within this network is crucial for developing breakthrough therapeutic strategies.

## 6 Therapeutic strategies: from pharmacological interventions to Traditional Chinese Medicine applications

### 6.1 Targeting macrophage polarization and the inflammasome

Beyond endogenous immune regulatory mechanisms, exogenous pharmacological interventions have emerged as a potential strategy for promoting nerve regeneration. A central therapeutic strategy is to shift the balance from pro-inflammatory M1 to pro-reparative M2 macrophages. This can be achieved by targeting key regulatory hubs. The NLRP3 inflammasome is a critical driver of M1 polarization. The natural alkaloid berberine (BBR) has been shown to promote functional recovery in a sciatic nerve injury model by directly inhibiting NLRP3 activation and IL-1β maturation (Godai and Moriyama, 2022; Wang J. et al., 2024). This inhibition skews macrophages towards an M2 phenotype, characterized by increased expression of Arg1 and IL-10, creating a microenvironment conducive to Schwann cell-mediated repair (Chew et al., 2013; Elsayed et al., 2020). Similarly, the diabetes drug metformin exerts immunomodulatory effects *via* activation of AMP-activated protein kinase (AMPK), which inhibits the NF-κB pathway and promotes M2 marker expression (Postler et al., 2021). Table 2 lists several common nerve repair drugs.

**TABLE 2:** Research on drugs promoting peripheral nerve regeneration through immunomodulation.

Drug	Primary target immune cells	Key factors/Pathways involved	Disease/Injury model studied
Berberine	Macrophages/Microglia	Inhibits NLRP3 inflammasome assembly/activation; downregulates M1 markers (iNOS, IL-1β); upregulates M2 markers (Arg1, IL-10)	Sciatic nerve injury model
Metformin	Macrophages/Microglia	AMPK pathway; inhibits NF-κB; promotes M2 polarization	Sciatic nerve crush injury, diabetic peripheral neuropathy
Fasudil	Macrophages/Neutrophils	RhoA/ROCK pathway; reduces neutrophil infiltration; promotes M2 polarization	Sciatic nerve transection injury
Rapamycin	Macrophages, T cells	mTOR pathway; modulates macrophage autophagy and polarization	Sciatic nerve injury, chemotherapy-induced peripheral neuropathy
Curcumin	Macrophages/Microglia	Inhibits TLR4/NF-κB and NLRP3 inflammasome pathways	Sciatic nerve chronic constriction injury, diabetic neuropathy
Resveratrol	Macrophages/Schwann cells	SIRT1 pathway; inhibits release of inflammatory factors	Sciatic nerve injury
Minocycline	Macrophages/Microglia	Inhibits MMP-9; reduces pro-inflammatory cytokines (TNF-α, IL-1β)	Sciatic nerve injury, neuropathic pain model
NSAIDs (e.g., celecoxib)	Macrophages	Inhibits COX-2; reduces inflammatory mediators like prostaglandins	Sciatic nerve injury (effects are controversial, dependent on timing and dosage)
Fingolimod	T cells, B cells	Sphingosine-1-phosphate receptor modulator; sequesters lymphocytes in lymph nodes	Sciatic nerve injury, autoimmune neuritis

### 6.2 Modulating the inhibitory scar environment

Another approach is to dismantle the inhibitory barriers to regeneration. CSPGs in the glial scar activate the growth cone-collapsing RhoA/ROCK pathway. The ROCK inhibitor fasudil has demonstrated efficacy in nerve injury models by not only reducing this CSPG-mediated inhibition but also by decreasing neutrophil infiltration and promoting M2 macrophage polarization, thereby acting on both the chemical and cellular barriers to regeneration (Ouyang et al., 2024).

### 6.3 The multi-target regulatory advantages of Traditional Chinese Medicine

Based on an in-depth understanding of the inflammatory and immune mechanisms underlying neuropathy, therapeutic strategies targeting these processes have become a research focus. Among them, Traditional Chinese Medicine (TCM) compounds exhibit unique potential due to their multi-component, multi-target characteristics (Yang et al., 2025). Taking the JinMaiTong (JMT) compound, used for treating DPN, as an example, its formulation follows the TCM principles of “tonifying the kidney, activating blood circulation, warming the tendons, and unblocking the collaterals,” targeting the core pathogenesis of DPN identified in TCM as “kidney deficiency and blood stasis” (Bai et al., 2021). Clinical studies have preliminarily confirmed its ability to improve symptoms and nerve conduction velocity in DPN patients (Hu et al., 2025).

To elucidate its scientific basis, a series of fundamental studies have been conducted from the whole-animal level down to the cellular level. Research indicates that JMT not only ameliorates glucolipid metabolism and reduces oxidative stress and apoptosis but also upregulates the expression of neurotrophic factors (such as NGF and CNTF) in the sciatic nerve (Maisonpierre et al., 1990; Yin et al., 2015). Most importantly, subsequent research has further focused on inflammatory pathways. Utilizing modern molecular biology techniques, it has been confirmed that JMT likely alleviates inflammatory damage in nerve tissue, promotes Schwann cell proliferation, and enhances their neurotrophic function by regulating key signaling pathways such as NF-κB. Consequently, it exerts a synergistic effect promoting nerve repair and regeneration through multiple targets and pathways (Molina-Gonzalez et al., 2023). The research paradigm established by JMT provides valuable clues for discovering drugs that treat peripheral neuropathy through immune regulation from the repository of traditional medicine. It is important to note that while preclinical studies and preliminary clinical trials of several months’ duration support the safety and efficacy of JMT for DPN, comprehensive long-term toxicological and pharmacokinetic data from large-scale, multi-year human studies are still limited. Future research adhering to modern drug development standards is needed to fully establish its long-term safety profile and optimal dosing regimens, which is a crucial step for its broader clinical translation and acceptance.

### 6.4 Cellular therapies: harnessing and engineering cells for immunomodulation

Beyond pharmacological agents, cellular therapies have emerged as a potent strategy to directly deliver or instruct immunomodulatory cells to the injury site, offering a dynamic approach to reshape the regenerative microenvironment (Petrus-Reurer et al., 2021). The core principle is to leverage the innate abilities of certain cell types to modulate macrophage polarization from a pro-inflammatory (M1) to an anti-inflammatory, pro-regenerative (M2) phenotype, thereby resolving chronic inflammation and creating a conducive environment for repair.

Among the most extensively investigated candidates are mesenchymal stem/stromal cells (MSCs). Transplanted MSCs secrete a wide array of bioactive factors—such as PGE2, TGF-β, and IL-10-that collectively suppress pro-inflammatory M1 macrophage activation while promoting their polarization toward an M2 phenotype (Liu et al., 2019). This shift is crucial for dampening chronic inflammation, reducing fibrosis, and enhancing Schwann cell-mediated repair and axonal regeneration. The efficacy of this approach is not limited to direct cell-cell contact; MSC-conditioned media (MSC-CM), containing the paracrine secretome of these cells, has been shown to significantly inhibit the expression of pro-inflammatory mediators (e.g., iNOS, COX-2, IL-1β, IL-6) in macrophages by suppressing key signaling pathways like NF-κB and MAPK (Chang et al., 2021). Furthermore, a novel and sophisticated evolution of this concept involves using MSC-derived extracellular vesicles (MSC-EVs). These vesicles encapsulate therapeutic cargo (e.g., miRNAs, proteins) and can efficiently deliver it to target cells, including macrophages, to instruct an M2-polarized state. MSC-EVs offer advantages over whole-cell therapies, including reduced risks of tumorigenicity and immunogenicity, and easier storage and standardization.

Other innovative strategies involve the direct administration of pre-polarized immune cells. This includes the transfusion of ex vivo-generated regulatory macrophages (M2-like) or the modulation of regulatory T cells, which can subsequently influence endogenous macrophage populations at the injury site towards a reparative phenotype (Cai et al., 2021). These approaches aim to “reset” the local immune landscape more rapidly and precisely than systemic drug administration.

The future of cellular therapy lies in combination and bioengineering strategies. This includes integrating MSCs or engineered macrophages within biomaterial scaffolds that provide structural guidance and controlled release of supportive factors, or creating “smart” cells *via* gene editing to respond to specific inflammatory cues within the nerve lesion. While challenges related to cell source, survival, delivery, and precise control of function remain, cellular therapies represent a paradigm shift towards leveraging the body’s own regulatory systems for precise, multifaceted immune modulation in nerve repair.

## 7 Current challenges and research controversies

Despite a deepening understanding of the mechanisms by which inflammation influences peripheral nerve regeneration, its translation into clinical practice faces significant challenges and important academic controversies.

The primary challenge lies in the precise spatiotemporal control of the inflammatory response. Inflammation plays diametrically opposed roles at different stages following injury: the acute inflammatory response is indispensable for clearing debris and initiating repair, whereas persistent inflammation in the chronic phase is markedly detrimental (Eming et al., 2007). However, with current technological limitations, it is difficult to precisely distinguish between these stages and intervene accordingly in a clinical setting. Consequently, strategies aimed at suppressing inflammation risk simultaneously interfering with its essential reparative functions (de la Garza-Kalife et al., 2025).

Secondly, controversy persists regarding the dual nature of inflammatory cytokines (Popa and Popa, 2021). Although *in vitro* studies have confirmed the beneficial effects of specific cytokines under certain conditions, most *in vivo* research, particularly in chronic disease models like diabetic peripheral neuropathy, predominantly observes their damaging effects. This discrepancy suggests that the beneficial actions of inflammatory cytokines are highly dependent on their local concentration, duration of action, and the specific microenvironmental context (Shachar and Karin, 2013). Within the complex *in vivo* milieu, these conditions are difficult to replicate or control, explaining why it is challenging to capture and harness their beneficial effects at the whole-animal or clinical level. This also highlights the significant gap between simplistic *in vitro* systems and the complexity of living organisms.

Finally, individual variability and targeted delivery represent core obstacles to clinical translation. In populations such as individuals with diabetes, the inflammatory response itself is more persistent and intense, and there are individual differences in treatment response. Furthermore, systemic administration of anti-inflammatory drugs may cause broad immunosuppression, increasing the risk of infection (Coutinho and Chapman, 2011). Therefore, developing delivery strategies that can precisely target immune cells at the injury site and achieve localized, controllable modulation-rather than systemic intervention-is a critical challenge that future research must overcome (Alsaiari et al., 2025).

## 8 Future perspectives and research directions

Building upon an in-depth understanding of inflammatory mechanisms, therapeutic strategies for peripheral nerve regeneration are shifting from single-target approaches toward multi-pathway, dynamically regulated precision interventions. Core strategies include: modulating macrophage polarization states using pharmacological agents (e.g., IL-4, statins) or inhibitors (e.g., MCC950) to balance the immune microenvironment; applying chondroitin’s ABC to degrade inhibitory scar components or using neutralizing antibodies against TGF-β to reduce physical barriers; and utilizing biomaterial scaffolds for the local sustained release of neurotrophic factors (e.g., BDNF, GDNF) or combined with gene transfection techniques to provide continuous nutritional support for neurons.

Future research will focus on leveraging cutting-edge technologies such as single-cell sequencing and spatial transcriptomics to deeply analyze the heterogeneity and dynamic interaction networks of local immune cells and glial cells at the injury site. Concurrently, exploring novel mechanisms like metabolic reprogramming and neuro-immune crosstalk will provide fresh perspectives for understanding nerve regeneration. In the realm of translational medicine, a major future direction involves developing intelligent, responsive biomaterials capable of releasing immunomodulatory factors or neurotrophic factors in real-time based on microenvironmental changes, thereby achieving spatiotemporally specific and precise regulation of the regenerative microenvironment. The distinctive advantages of Traditional Chinese Medicine (TCM) compounds in multi-target regulation will also be further explored and scientifically validated, offering more options for the treatment of peripheral nerve injuries.
